# Macrophage-Targeting Gene Delivery Using a Micelle Composed of Mannose-Modified Lipid with Triazole Ring and Dioleoyl Trimethylammonium Propane

**DOI:** 10.1155/2015/350580

**Published:** 2015-10-05

**Authors:** Ichiki Fukuda, Shinichi Mochizuki, Kazuo Sakurai

**Affiliations:** ^1^Department of Chemistry and Biochemistry, The University of Kitakyushu, 1-1 Hibikino, Wakamatsu-ku, Kitakyushu, Fukuoka 808-0135, Japan; ^2^NexTEP, Japan Science and Technology Agency, 4-1-8 Honcho, Kawaguchi, Saitama 332-0012, Japan

## Abstract

Gene carriers with cell specific ligand molecules are needed for the treatment of several diseases. Mannose is known to be recognized and incorporated into the cells through mannose recognition lectins that are exclusively expressed on macrophages. In this study, we synthesized two types of mannose-modified lipids with different stereoisomer (*α*-mannose and *β*-mannose). To make a complex with plasmid DNA (pDNA), termed “lipoplex,” we prepared a two-component micelle made from cationic lipid; dioleoyltrimethylammoniumpropane (DOTAP); and mannose-modified lipid (D/*α*-Man or D/*β*-Man). The prepared D/*α*-Man lipoplexes were able to bind to one of the *α*-mannose lectins concanavalin A (ConA) immobilized on gold substrate in the quartz-crystal microbalance sensor cell. D/*β*-Man lipoplexes did not show any frequency changes. These results indicate that the mannose residues were exposed on the lipoplexes, leading to not only the binding to ConA but also the prevention of nonspecific interactions with proteins. Both lipoplexes showed high transfection efficiencies to RAW264.7 cells which have several kinds of mannose lectins. This delivery system to macrophages may overcome the problems for gene therapy and may be used for the treatment of immune diseases involved in macrophages.

## 1. Introduction

The development of targeted cellular gene delivery systems is an important research theme for clinical applications of gene therapies. Although nonpathogenic viral vectors such as retroviruses and lentiviruses are mainly used, nonviral alternatives have been studied because of their advantages of safety and low manufacturing costs [1]. The most commonly used synthetic gene carriers are cationic lipids and polymers, which can form complexes with negatively charged plasmid DNA (pDNA) via electrostatic interactions. One of the greatest obstacles to overcome before they can be used in human therapy is their low uptake efficiency into the target cells because of a lack of cellular selectivity. To this end, cationic carriers are modified with various compounds to improve pDNA accumulation in the target cells. A typical example is attaching a carbohydrate as a ligand molecule. Some cells express lectins, which are carbohydrate-binding proteins that exhibit high specificity for sugar molecules. The relationships between carbohydrates and cells with lectin have been exploited for cell-specific drug delivery, such as galactose for hepatocytes [2–5], hyaluronic acid for liver sinusoidal endothelial cells [6, 7], and mannose for macrophages [8, 9].

Macrophages are immune cells that play an important role in immune system regulation. As proinflammatory cytokines secreted by macrophages are related to the pathogenesis of various inflammatory diseases such as acute hepatitis [10] and ulcerative colitis [11, 12], inhibiting such cytokines is beneficial for patients. Mannose-recognizing lectins are exclusively expressed on macrophages; they recognize glycoproteins with mannose,* N*-acetylglucosamine, and fucose residues and subsequently internalize them [13, 14]. Several studies have focused on mannose residues as a pilot molecule for targeting macrophages.

We previously reported a series of lipids with an aromatic linker connected with amine or amidine that can be used as transfection reagents with better efficiency and lower cytotoxicity than conventional reagents [15, 16]. Analysis of the hydrophobic moieties that induce high transfection efficiency by altering the alkyl chain lengths and the positions revealed that lipids with an amino residue attached to C12 in the meta-meta position exhibit the highest efficiencies [16]. In addition, lipids can form stable micelles at low concentrations as a result of hydrophobic interactions among aromatic rings and 2 alkyl chains as well as *π*-*π* stacking derived from aromatic rings. After synthesizing galactose-modified lipids, evaluation of the transfection efficiencies for hepatocytes with asialoglycoprotein receptor (ASGPR), which recognizes galactose and* N*-acetylgalactosamine residues on glycoproteins, revealed that the complexes comprising galactose-modified lipids, cationic lipids, and pDNAs exhibited ASGPR-dependent gene expression [5].

In the present study, we synthesized mannose-modified lipids with an aromatic ring with C12 in the meta-meta position. The mannose-modified lipids were mixed with cationic lipids to form complexes with DNA, termed “lipoplexes,” and their transfection efficiencies in macrophages were evaluated.

## 2. Materials and Methods

### 2.1. Materials

3,5-Dihydroxybenzaldehyde, potassium carbonate, propargyl bromide, and RPMI-1640 medium were purchased from Wako Pure Chemical Industries, Ltd., (Osaka, Japan). 1-Bromododecane, sodium borohydride, sodium hydride, 2,3,4,6-tetra-O-benzoly-D-mannopyranose, trifluoromethanesulfonic anhydride, diphenyl sulfoxide, N,N-dimethylformamide, trimethylsilyl azide, and 2-chloro-1,3-dimethylimidazolium chloride were purchased from Tokyo Chemical Industry Co., (Tokyo Japan). Sodium methoxide was purchased from Kanto Chemical Co., (Tokyo Japan). Copper(II)sulfate pentahydrate was purchased from Sigma-Aldrich (St. Louis, MO).

### 2.2. Synthesis of the Mannose-Modified Lipid (Scheme 1)

2,3,4,6-Tetra-O-benzoly-D-mannopyranose and diphenyl sulfoxide were mixed in solution of dichloromethane and toluene (dichloromethane : toluene = 1 : 3) at molar ratio of 1 : 1 and stirred at −78°C for 10 minutes. The reactant was added to trifluoromethanesulfonic anhydride at molar ratio of 1 : 1.3 and stirred at −45°C for 30 minutes. The reactant was added to trimethylsilyl azide at molar ratio of 1 : 5 and stirred at −45°C for 30 minutes, followed by −20°C for 2.5 hours [17]. The reaction mixture was extracted with ethyl acetate and purified compound I by silica gel chromatography using a mixture of ethyl acetate and hexane (ethyl acetate : hexane = 1 : 3) as a mobile phase.

2,3,4,6-Tetra-O-benzoly-D-mannopyranose, 2-chloro-1,3-dimethylimidazolium chloride, sodium azide, and triethylamine were mixed in solution of N,N-dimethylformamide at molar ratio of 1 : 10 : 10 : 3 and stirred at 0°C for 2 hours [18]. The reaction mixture was extracted with ethyl acetate and purified compound II by silica gel chromatography using a mixture of ethyl acetate and hexane (ethyl acetate : hexane = 2 : 3) as a mobile phase.

**Scheme 1:**
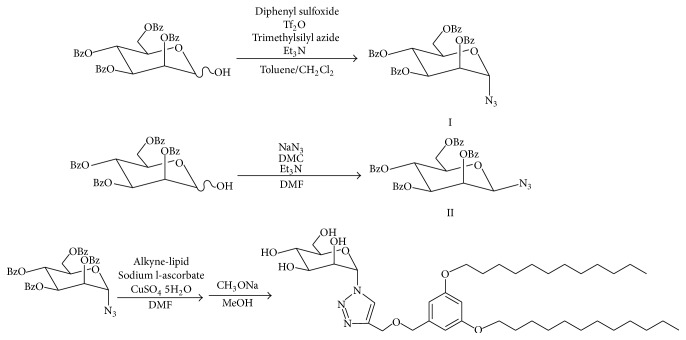
Synthetic route of *α*-mannose- or *β*-mannose-modified lipid through click chemistry.

The compound I or compound II was an attached aromatic ring with C12 in the meta-meta position as shown in Scheme 1 in a similar manner to our previous report [5]. The obtained mannose-modified lipid was identified by ^1^H NMR.

### 2.3. Preparation of the Lipoplex Composed of Mannose-Modified Lipid/Cationic Lipid Micelle and pDNA

We mixed the mannose-modified lipid and dioleoyltrimethylammoniumpropane (DOTAP; Sigma-Aldrich) at the same molar ratio and dissolved them in chloroform and vacuum dried. Each mixture was dissolved in water and added to the pDNA encoding luciferase (pGL3-Control Vector; Promega, Madison, WI) at the indicated N/P ratios (i.e., the cation/anion charge ratio, [cationic amino group]_DOTAP_/[anionic phosphate group]_nucleic acid_) and incubated for 1 hour. To confirm the complexation, the mixtures were separated by 1% agarose gel electrophoresis. DNA was stained with ethidium bromide and the image was obtained using a PharosFX (Bio-Rad, Richmond, CA).

### 2.4. *ζ* Potential and Size Measurements

We prepared the lipoplexes at indicated N/P ratios in 150 mM NaClaq, where we fixed DOTAP concentration at 0.3 mM. The zeta potentials and hydrodynamic radiuses were measured with a Nano-ZS (Malvern Instruments, Malvern, UK) at 25°C. The refractive index was 1.59.

### 2.5. Small-Angle X-Ray Scattering (SAXS) Measurement

SAXS measurements from the lipoplexes were carried out at BL40B2 SPring-8 with a 0.7 m camera using a Rigaku imaging plate (30 × 30 cm, 3000 × 3000 pixels) as a detector. The wavelength of the beam was 1.0 Å, and the exposure time was 300 seconds. The obtained two dimensional image was circularly averaged to give an intensity *I*(*q*) versus *q* plots, where *q* is the magnitude of the scattering vector defined by *q* = 4*π*sin⁡*θ*/*λ* with the scattering angle of 2*θ*. The concentration of mannose-modified lipids was 3 mM.

### 2.6. Interaction between Concanavalin A (ConA) and Lipoplexes

ConA (Wako) was immobilized on gold substrate in the quartz-crystal microbalance (QCM) sensor cell (AFFINIX QN *μ*; INITIUM, Inc., Tokyo, Japan) at 10 *μ*g/mL for 1 h at r.t. After blocking with 1% BSA in PBS for 1 h, the sensor cell was filled with 500 *μ*L of 10 mM HEPES containing 2 mM CaCl_2_ adding the samples at 36 *μ*g/mL and measuring the frequency changes at 25°C.

### 2.7. Gene Transfection

RAW264.7 cells were seeded at 2.0 × 10^4^ cells in a 96-well microplate and incubated at 37°C under 5% CO_2_. The cells were cultured in RPMI-1640 containing 10% FBS and 100 U/mL penicillin and 0.1 mg/mL streptomycin. After 24 hours, the cells were transfected with the pDNA at 0.2 *μ*g/mL using the lipoplexes or Lipofectamine 2000 (Invitrogen, Carlsbad, CA). In brief, on the day of transfection, the wells were replaced with fresh medium without serum and added the lipoplexes at the indicated N/P ratio. After 6 hours, the wells were replaced with fresh medium containing serum. After 48 hours, the cells were washed with PBS twice adequately and then lysed with a lysis buffer from the luciferase assay kit (Promega). After adding luciferin, the luciferase activity in an aliquot of the cell lysate was measured with a luminescence plate reader (Wallac 1420; Perkin Elmer, Wellesley, MA). The protein concentration of each well lysate was determined with a standard protein assay (Dojindo, Kumamoto, Japan). The luciferase activity in each sample was normalized to the luminescence intensity per microgram of protein.

## 3. Results

### 3.1. Lipoplex Preparation and Characterization

**Figure 1:**
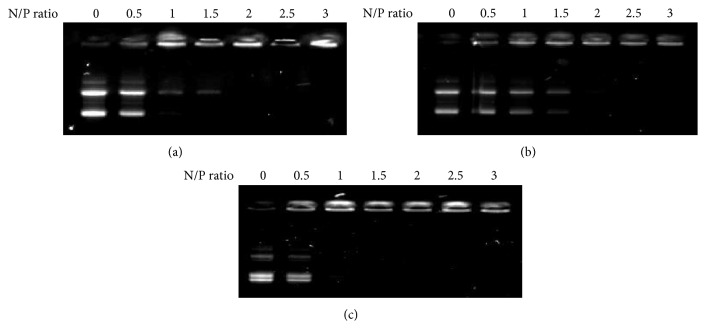
Confirmation of the complexation between pDNA and D/*α*-Man (a), D/*β*-Man (b), or DOTAP (c) micelles at indicated N/P ratios.

As the 2 prepared kinds of mannose-modified lipids (Scheme 1) have no cationic charge, they are unable to form a complex with pDNA via electrostatic interactions. In addition, the mannose-modified lipids themselves cannot form micelles in aqueous solution owing to their low solubility. In our previous study, we added the cationic lipid DOTAP to sugar-modified lipids with the same features to compensate for the deficits described above [5]. In fact, the mixture of DOTAP with sugar-modified lipid was dispersed in aqueous solution, forming lipoplexes with pDNA. Therefore, we added DOTAP to mannose-modified lipid at the same molar ratio; thus, we prepared binary micelles comprising DOTAP and *α*-mannose- or *β*-mannose-modified micelles, which were designated D/*α*-Man and D/*β*-Man, respectively. After mixing pDNA with D/*α*-Man, D/*β*-Man, or DOTAP at the indicated N/P ratios, lipoplex formation was examined by agarose gel electrophoresis (Figure 1). With DOTAP, there were no free pDNA bands observed at N/P > 2. The same results were obtained for the mixtures of pDNA with D/*α*-Man and D/*β*-Man micelles, indicating that the addition of mannose-modified lipids does not disturb lipoplex formation or galactose- or glucose-modified lipids. Therefore, D/*α*-Man and D/*β*-Man form stable complexes at N/P > 2.

**Figure 2:**
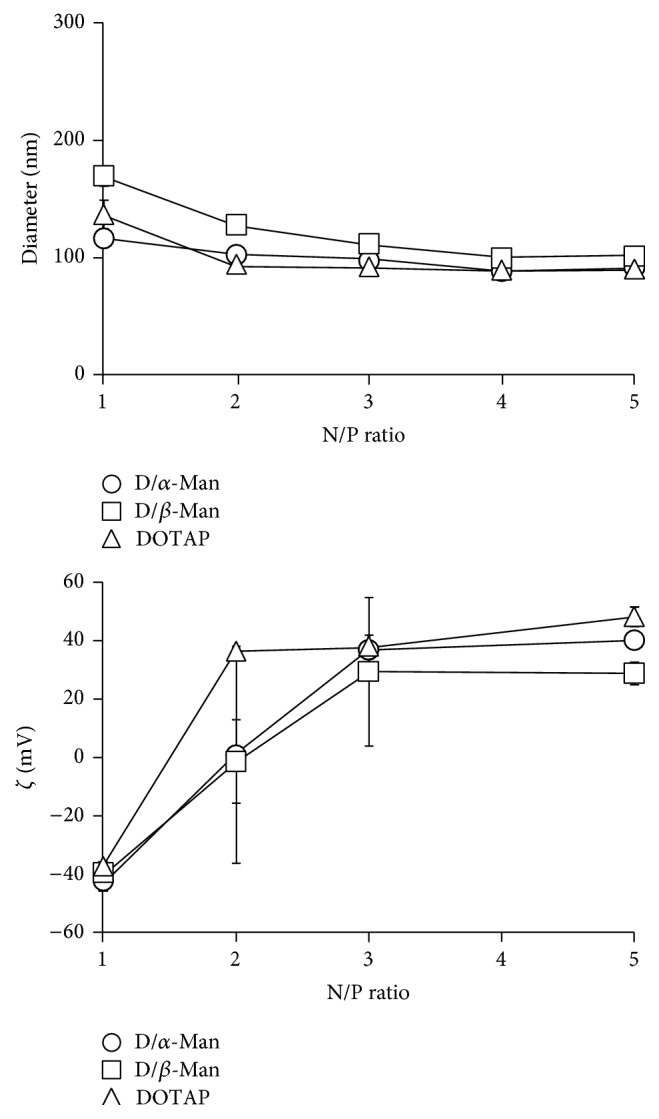
Diameters and *ζ* potentials for the lipoplexes determined with dynamic light scattering measurements.

The hydrodynamic radii and *ζ* potentials of the resultant lipoplexes were measured at various N/P ratios. The hydrodynamic radii were almost all ~100 nm and they exhibited a small polydispersity at all N/P ratios (Figure 2 and Supplementary Table  1 available online at http://dx.doi.org/10.1155/2015/350580). Supplementary Figure  1 shows the histograms of the lipoplexes at N/P = 2. All lipoplexes exhibited a single peak. These results indicate that the lipoplexes were dispersed without forming large aggregates. The *ζ* potentials of all samples increased with the increasing N/P ratio, plateauing at N/P = 3. The *ζ* potentials of all micelles exhibited around −40 mV (data not shown), which is consistent with the plateaued values. In particular, they changed drastically from a negative to a positive charge between N/P 1 and 2. The positive charge at N/P = 2 means that all pDNA was covered by cationic compounds, which is consistent with the complete complex formation at N/P = 2 (Figure 1). The *ζ* potentials of D/*α*-Man and D/*β*-Man lipoplexes were lower than those of DOTAP lipoplexes at N/P = 2 even at the same concentration of DOTAP. The *ζ* potential is related to the electrical charge at the interface between a solid surface and its liquid medium and does not reflect the total charge of the entire particle [19]. Therefore, these results suggest that both lipoplexes express the mannose residues on the surface, resulting in low *ζ* potential. The high positive *ζ* potentials at N/P > 3 can be attributed to the excessive feed of D/*α*-Man and D/*β*-Man micelles against pDNA. As high *ζ* potential might lead to nonspecific cellular uptake because of the electrostatic interaction between cellular membranes and lipoplexes, we used lipoplexes at N/P = 2 for all subsequent examinations.

**Figure 3:**
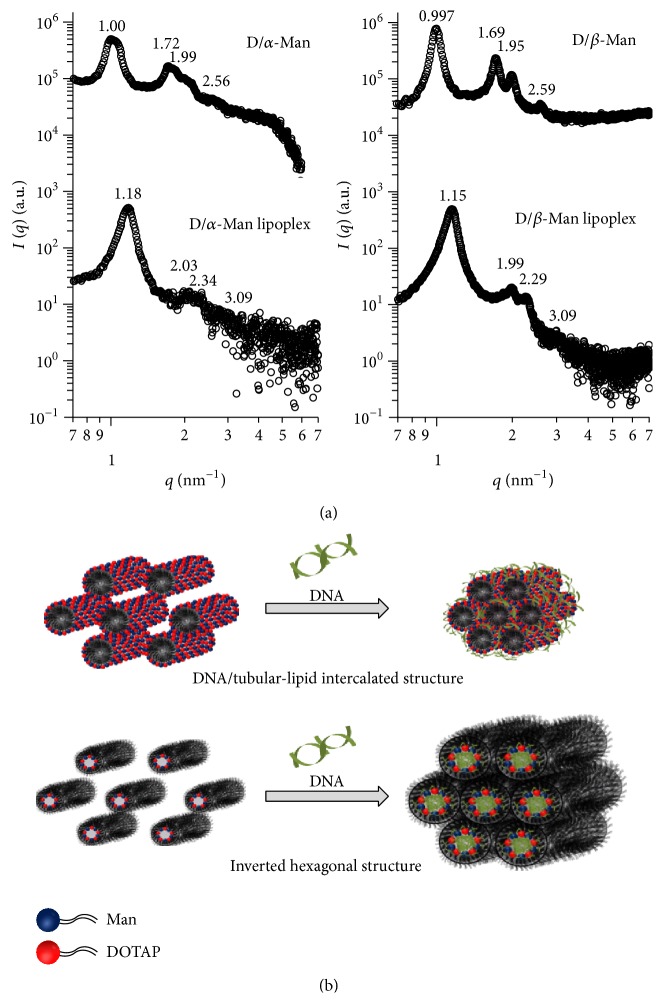
SAXS profiles of D/*α*-Man and D/*β*-Man micelles before and after complexation with pDNA at N/P = 2 (a) and the schematic illustration showing the structure of D/*α*-Man and D/*β*-Man lipoplexes (b).

Safinya et al. propose a relationship between the supramolecular structure of lipoplexes and their transfection efficiency with use of SAXS [20–23]. They state that the addition of pDNA causes most cationic lipids to undergo structural transition and that some form hexagonally packed cylinders including inverted hexagonal [21] and DNA/tubular-lipid intercalated structures [22]. Therefore, we examined the structures of D/*α*-Man and D/*β*-Man before and after complexation with pDNA by using SAXS (Figure 3(a)). All samples exhibited sharp diffraction peaks, indicating the formation of ordered structures. The positions satisfied the relation of 1 : $\sqrt{3}$ : 2 : $\sqrt{7}$, indicating that the micelles and lipoplexes formed a hexagonally packed cylindrical structure [24]. For both types of micelles, the addition of pDNA did not alter hexagonal packing, but the peak positions were shifted to the wider angle side. The intercylinder distances determined from the peak positions for D/*α*-Man, D/*β*-Man, D/*α*-Man lipoplexes, and D/*β*-Man lipoplexes were 7.25, 7.28, 6.15, and 6.31 nm, respectively. These results indicate that negatively charged pDNA reduces or cancels electrostatic repulsions between adjoining tubular lipids through intercalation, resulting in contracted cylinder distance; this implies that both lipoplexes adopt a DNA/tubular-lipid intercalated packing (Figure 3(b)).

In summary, at N/P = 2, D/*α*-Man, and D/*β*-Man, lipoplexes have the same characteristics including particle size, surface charge, and inner structure in which tubular lipids and pDNA are hexagonally packed within the particles.

### 3.2. Binding of D/*α*-Man and D/*β*-Man Lipoplexes to ConA

**Figure 4:**
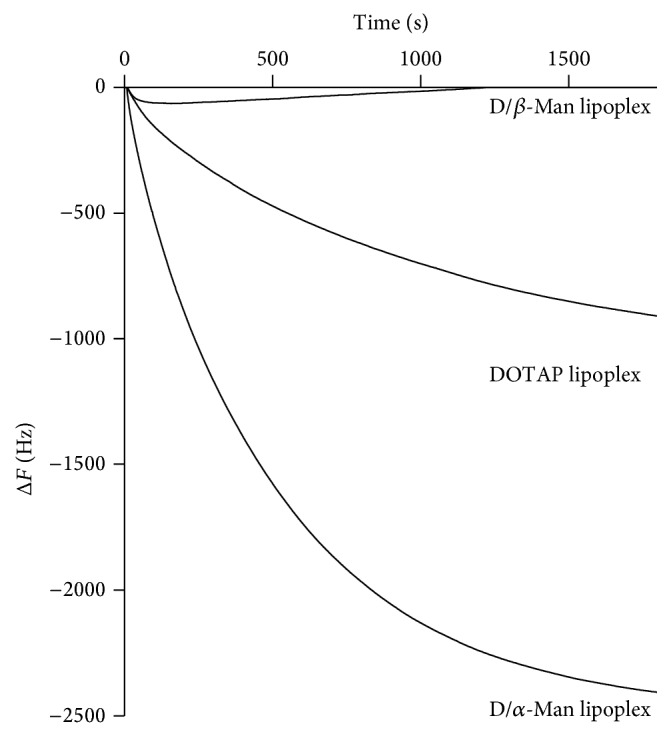
Time courses of frequency changes of ConA immobilized QCM in response to addition of the lipoplexes (N/P = 2).

We previously reported that lipoplexes comprising galactose-modified lipids, DOTAP, and pDNA bound to ASGPR resulted in strong gene expression in HepG2 cells containing ASGPRs. In the present study, we examined the binding of D/*α*-Man and D/*β*-Man lipoplexes to mannose-recognition proteins with QCM. QCM measures the decrease in the frequency of a quartz-crystal immersed in solutions, which is directly related to the increase in mass due to the surface adsorption of guest molecules onto the resonator. ConA was used as a model protein because it is a lectin that recognizes *α*-d-mannosyl and *α*-d-glucosyl groups [25].  Figure 4 shows that the QCM frequency changes when equal masses of D/*α*-Man, D/*β*-Man, or DOTAP lipoplexes were added to the QCM cell. The addition of D/*α*-Man lipoplexes rapidly decreased the frequency, whereas the addition of D/*β*-Man lipoplexes did not. The addition of DOTAP lipoplexes slightly decreased the frequency, albeit a smaller extent than D/*α*-Man lipoplexes. DOTAP lipoplexes, which have strong cationic characteristics, are considered to nonspecifically bind or adsorb to ConA or bovine serum albumin coated on the sensor cell via electrostatic interactions. These results indicate that the mannose moieties in the lipoplexes are exposed to the surface. *β*-mannose on the lipoplexes shields the cationic characteristic of DOTAP and prevents nonspecific binding, whereas *α*-mannose not only exhibited the same effect as *β*-mannose but also was definitely recognized and bound to the binding site.

### 3.3. Gene Expression Efficiency

**Figure 5:**
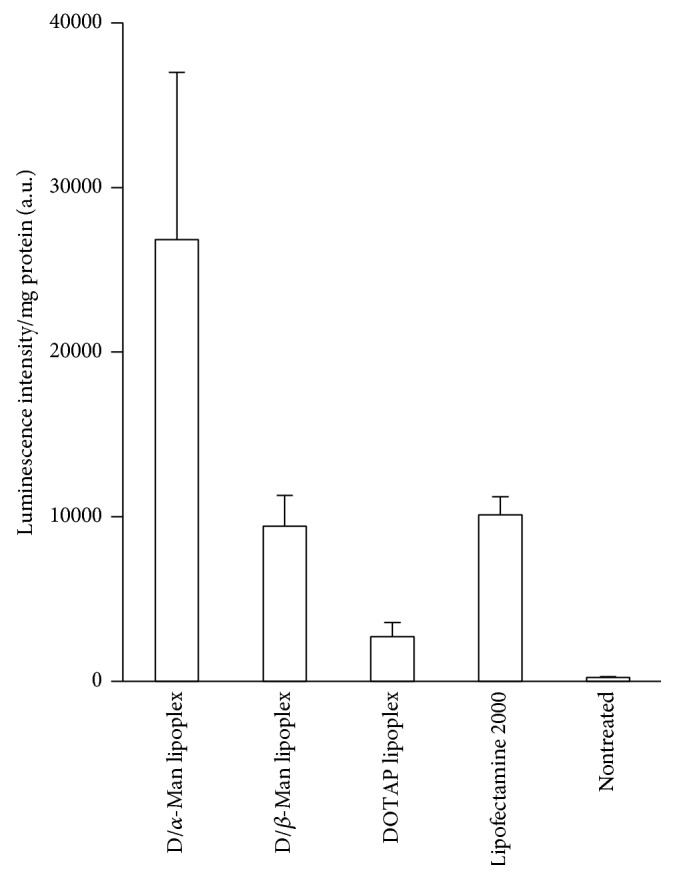
Transfection efficiencies for the lipoplexes at N/P = 2. Data represent the mean ± S.D. of triplicate wells.

We subsequently examined gene expression efficiency in murine macrophage RAW264.7 cells. D/*α*-Man lipoplexes induced the highest gene expression, which was 3 times higher than that induced by Lipofectamine 2000 (Figure 5). D/*β*-Man lipoplexes induced gene expression to the same level as that of Lipofectamine 2000. Meanwhile, DOTAP lipoplexes induced much lower gene expression than D/*α*-Man and D/*β*-Man lipoplexes. These results suggest that D/*α*-Man and D/*β*-Man lipoplexes can induce gene expression in macrophages with the aid of mannose-modified lipid; in particular, the gene expression was strongly dependent on *α*-mannose compared to *β*-mannose.

## 4. Discussion

Carriers with cell-specific ligand molecules can be critical for efficient targeted gene delivery. Because sugar is guaranteed to have high specificity for its particular lectin, sugar modification is a useful strategy for the abovementioned purpose. Furthermore, for some lectins, the affinity of their ligands increases with the valence of sugar residues. Lee et al. demonstrate that multivalent glycosides simultaneously bind to receptors, enhancing affinity in comparison to monovalent glycosides [26, 27]; this phenomenon is known as the “cluster effect.” Only cationic lipids or polymers can be used to bind pDNA to carriers. The N/P ratio is one of the factors that determine transfection efficacy. Although the lipoplexes with a high N/P ratio can easily interact with cellular membranes, they can simultaneously exhibit strong cellular toxicity. Lipoplexes with sugars on their surface can be used to resolve this dilemma, because they can be recognized by lectins even at low N/P ratios.

**Figure 6:**
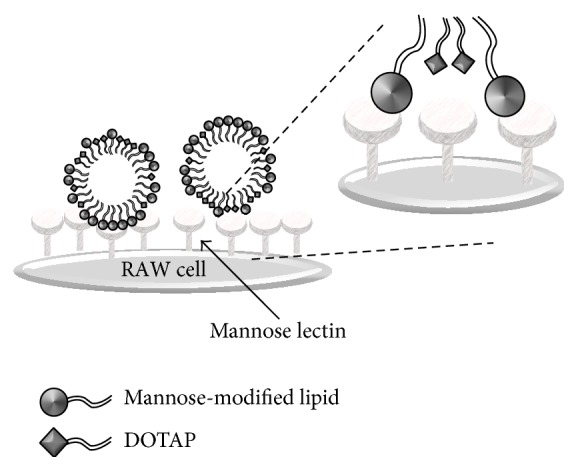
The schematic illustration showing the configurational relationship between mannose-modified lipids and DOTAP in the micelles.

We prepared 2 types of stereoisomeric mannose-modified lipids as ligands for mannose lectin and generated micelles by mixing DOTAP for pDNA binding. The lipoplexes bound to the mannose lectin ConA (Figure 4) and induced high transfection efficiency in RAW264.7 cells (Figure 5). These results strongly indicate that mannose residues can be exposed to the surface of lipoplexes. Considering its molecular structure, DOTAP bends greatly because of the* cis*-double bonds in the alkyl chains. The linear length of DOTAP is shorter than that of mannose-modified lipids. Therefore, most DOTAP is considered to be located internally in the micelles (Figure 6), decreasing the *ζ* potential of D/*α*-Man and D/*β*-Man lipoplexes compared to that of DOTAP lipoplexes (Figure 2) as well as preventing nonspecific proteins interactions (Figure 4).

We prepared micelles by mixing mannose-modified lipids and DOTAP at the same molar ratio. Fortunately, the lipoplexes exhibited mannose-dependent gene expression, suggesting that the configurations between mannoses and their density on the lipoplexes are sufficient for recognition by lectins. Their configurations are mainly dominated by the mannose-modified lipid content in micelles and can be easily controlled by changing the mixing ratio between mannose-modified lipids and DOTAP. Therefore, it is possible to optimize lipoplexes for inducing further transfection efficiency.

Macrophages have several kinds of mannose lectins such as CD206 (mannose receptor) [28, 29], CLEC4E [30, 31], and CLEC12A [32, 33]. Although some lectins are known to mainly recognize *α*-mannose, fucose, and high mannose with a mixture of *α*- and *β*-linkages, the recognition of other lectins remains unknown [14]. RAW cells may express mannose lectins that preferentially recognize *α*-mannose, resulting in higher transfection efficiency of D/*α*-Man lipoplexes than D/*β*-Man lipoplexes (Figure 5).

In conclusion, lipoplexes comprising equimolar amounts of pDNA and D/*α*-Man exhibit good transfection efficiency at N/P = 2. Although D/*α*-Man and D/*β*-Man lipoplexes do not differ with respect to size, surface charge, or supramolecular structure, they distinctly differ with respect to ConA recognition, indicating the exposure of mannose residues on these lipoplexes. Both lipoplexes exhibited high gene expression, which may be dependent on the expression level of mannose lectins on RAW cells. Therefore, the present study suggests that our lipoplexes with mannose residues can be used in the treatment of macrophage-related diseases.

## Supplementary Material

Polydispersity index of the lipoplexes determined with dynamic light scattering measurements. . Histograms of diameters for the lipoplexes determined with dynamic light scattering measurements (N/P = 2).
