# Supplementary material for: Macrophage-Targeting Gene Delivery Using a Micelle Composed of Mannose-Modified Lipid with Triazole Ring and Dioleoyl Trimethylammonium Propane
Source: Biomed Res Int. 2015 Oct 5;2015:350580. doi: 10.1155/2015/350580 (PMC4609769; doi:10.1155/2015/350580)
Supplement: Supplementary file 1 — Polydispersity index of the lipoplexes determined with dynamic light scattering measurements. . Histograms of diameters for the lipoplexes determined with dynamic light scattering measurements (N/P = 2). [file 350580.f1.doc]

## Macrophage-targeting gene delivery using a micelle composed of mannose-modified lipid with triazole ring and dioleoyl trimethylammonium propane

Ichiki Fukuda^a^, Shinichi Mochizuki^a^, and Kazuo Sakurai^a,b,*^

^a^Department of Chemistry and Biochemistry, The University of Kitakyushu, 1-1, Hibikino, Wakamatsu-ku, Kitakyushu, Fukuoka, 808-0135, Japan.

^b^NexTEP, Japan Science and Technology Agency, 4-1-8, Honcho, Kawaguchi, Saitama 332-0012, Japan.

*Corresponding author. Department of Chemistry and Biochemistry, The University of Kitakyushu, 1-1, Hibikino, Wakamatsu-ku, Kitakyushu, Fukuoka, 808-0135, Japan.

E-mail address: sakurai@kitakyu-u.ac.jp

Table S1. Polydispersity index of the lipoplexes determined with dynamic light scattering measurements.

|  | N/P | | | | |
| --- | --- | --- | --- | --- | --- |
|  | 1 | 2 | 3 | 4 | 5 |
| D/α-Man lipoplex | 0.33 | 0.33 | 0.38 | 0.35 | 0.42 |
| D/β-Man lipoplex | 0.33 | 0.25 | 0.25 | 0.24 | 0.34 |
| DOTAP lipoplex | 0.44 | 0.33 | 0.37 | 0.39 | 0.43 |

Supplementary Figure 1

Figure S1. Histograms of diameters for the lipoplexes determined with dynamic light scattering measurements (N/P = 2).
